# Brain-age in ultra-low-field MRI: How well does it work?

**DOI:** 10.1162/IMAG.a.1352

**Published:** 2026-09-15

**Authors:** Francesca Biondo, Carly Bennallick, Sophie A. Martin, Lemuel Puglisi, Thomas C. Booth, David A. Wood, Juan Eugenio Iglesias, František Váša, James H. Cole

**Affiliations:** UCL Hawkes Institute, UCL, London, United Kingdom; Department of Neuroimaging, Institute of Psychiatry, Psychology and Neuroscience, King’s College London, London, United Kingdom; University of Catania, Catania, Italy; School of Biomedical Engineering & Imaging Sciences, King’s College London, London, United Kingdom; Department of Neuroradiology, Ruskin Wing, King’s College Hospital NHS Foundation Trust, London, United Kingdom; Martinos Center for Biomedical Imaging, Massachusetts General Hospital and Harvard Medical School, Charlestown, MA, United States; Computer Science and Artificial Intelligence Laboratory, Massachusetts Institute of Technology, Cambridge, MA, United States; Dementia Research Centre, UCL, London, United Kingdom

**Keywords:** low field MRI, brain-age, structural MRI, SynthSR, FreeSurfer, open data

## Abstract

Brain-age estimates the brain’s biological age from neuroimaging data and has been proposed as a biomarker of brain health and disease risk. While brain-age estimation commonly uses high-field (HF) magnetic resonance imaging (MRI) (>1.5 T), this is costly and inaccessible, limiting its applicability. Emerging ultra-low-field (ULF) MRI (<0.1 T) is cheaper and more accessible, but its lower resolution may limit the reliability of biomarkers such as brain-age. We assessed different brain-age pipelines in 23 adults scanned on one HF system (GE Signa Premier at 3 T) and two identical ULF systems (Hyperfine Swoop at 64 mT) located at two different sites, hereafter referred to as ULF1 and ULF2. We used 14 distinct acquisitions defined by T1- or T2-weighting, resolution, and preprocessing: raw anisotropic orientations (axial, coronal, sagittal), isotropic scans, and super-resolution derivatives from multi-resolution registration (MRR) and SynthSR. These inputs (a total of *n* = 573 scans) were analyzed with five brain-age software packages (BrainageR, SynthBA, MIDI, DeepBrainNet, PyBrainAge). Performance evaluation entailed validity (brain-age vs. actual age), correspondence (ULF brain-age vs. HF brain-age) and test-retest reliability (ULF1 brain-age vs. ULF2 brain-age). Overall, results were mixed across pipelines, although several ULF pipelines performed comparably to HF. The four best-performing combinations were SynthBA on T2 scans without SynthSR, MIDI on T2 scans without SynthSR, PyBrainAge on T1 scans with SynthSR and using FreeSurfer recon-all-clinical, and BrainageR on T1 scans with SynthSR. These showed moderate-to-strong validity (r=0.76 –0.92, $R^{2}\;=0.54$ –0.64, Mean Absolute Error [MAE] =6.7 –8.21 years), moderate-to-strong correspondence to HF (r=0.84 –0.93, Intraclass Correlation Coefficient [ICC] =0.72 –0.92), and excellent test-retest reliability (r=0.97 –0.99, ICC =0.97 –0.99). Moreover, some anisotropic acquisitions achieved comparable validity and reliability to MRR images when tested with the best-performing model, SynthBA ($R^{2}\;=0.57$ –0.62, ICC [CI] =0.99 [0.97–1.00], for coronal T2). This first systematic evaluation of brain-age at ULF demonstrates that accurate and reliable estimates can be achieved across multiple pipelines, without necessarily requiring image enhancement. Performance depended on the combination of model, scan type, and preprocessing. ULF brain-age estimation could be a practical and scalable tool for clinical decision-making, population research, and long-term patient monitoring, thereby helping to make advanced neuroimaging biomarkers more accessible worldwide.

## 1 Introduction

Magnetic resonance imaging (MRI) biomarkers remain a cornerstone of brain health research. Over the past decade, their translational utility has expanded rapidly, providing insights into brain structure, pathology, and treatment monitoring across neurological and psychiatric conditions (Thompson et al., 2020; Young et al., 2024) as well as brain changes and differences in healthy individuals, for example in normative ageing studies (Bethlehem et al., 2022; Dorfschmidt et al., 2025; Rutherford et al., 2022a, 2022b).

Despite these advances, MRI-derived markers are not without limitations. Conventional high-field (HF) MRI (1.5–3 T) systems remain largely inaccessible in many settings. This is largely due to high purchase and operational costs (e.g., cooling, trained staff) as well as complex installation requirements (e.g., shielding, structural reinforcement, upgraded power supply) (Arnold et al., 2023). Contraindications such as metal implants or claustrophobia further restrict their utility. These barriers lead to long waiting lists, limited access in low-resource settings, and suboptimal demographic representativeness in MRI research cohorts (Abate et al., 2024; Martin et al., 2023).

Recently, a new generation of portable ultra-low-field (ULF) MRI systems (<0.1 T ) has emerged, offering a compelling alternative to conventional scanners. These inexpensive systems have negligible operational costs (e.g., low power, minimal training to run), can be operated from a standard power outlet, used at the bedside, and deployed in settings where HF MRI is impractical (Arnold et al., 2023; Kimberly et al., 2023; Sorby-Adams et al., 2024; Váša et al., 2025). Their open designs improve patient comfort and tolerance, and their reduced field strength minimizes many common contraindications. These features make ULF MRI a promising avenue for increasing accessibility and broadening participation in neuroimaging research and clinical care. For example, their current deployment in low-to-middle-income countries for paediatric neurodevelopment research underlines their feasibility for large-scale global health studies (Abate et al., 2024; Pretzsch et al., 2025). In turn, increased accessibility and lower cost make repeated scans far more feasible, facilitating longitudinal tracking of brain changes for both clinical and research purposes.

However, ULF MRI has limitations, with lower signal-to-noise ratio (SNR) and lower resolution compared with HF MRI (Arnold et al., 2023; Váša et al., 2025). Nonetheless, early findings suggest that ULF MRI can provide clinically meaningful information (Arnold et al., 2022; Deoni et al., 2023; Sorby-Adams et al., 2024), and image denoising or enhancement techniques can help overcome its inherent resolution limitations (Deoni et al., 2022; Baljer et al., 2025a, 2025b; Briski et al., 2024; Iglesias et al., 2023). For example, multi-resolution registration (MRR) improves effective resolution and test-retest reliability (Deoni et al., 2022; Váša et al., 2025), while machine learning-based super-resolution techniques such as SynthSR (Iglesias et al., 2023) enable the synthesis of isotropic, high-resolution images from low-quality inputs, improving segmentation accuracy and agreement with HF scans. Notably, Sorby-Adams et al. (2024) demonstrated that hippocampal and white matter hyperintensity measures derived from ULF scans processed with SynthSR are highly comparable to those from HF MRI. In the context of brain-age estimation, Valdes-Hernandez et al. (2023) previously proposed using SynthSR to prepare clinical-grade MRIs for downstream brain-age prediction. SynthSR is integrated into the widely used neuroimaging preprocessing software FreeSurfer (Fischl, 2012; Iglesias et al., 2023).

In this study, we extend these advances to brain-age estimation, systematically evaluating this measure at ULF. Brain-age is an index of the brain’s biological age, typically obtained by training a machine learning model on MRI features from a healthy reference cohort to predict chronological age (Cole & Franke, 2017; Gaser et al., 2024). The trained model is then used to estimate age, or brain-age, in unseen scans. The difference between predicted and chronological age, commonly termed the brain-age delta or brain predicted age difference (brainPAD), serves as the key variable in downstream analyses. BrainPAD has emerged as a simple yet sensitive marker of brain health, providing clinically relevant information about both current status and future outcomes in patient and healthy cohorts, with older-than-expected brain-ages linked to outcomes such as cognitive decline, increased dementia risk, and psychiatric diagnoses such as schizophrenia (Biondo et al., 2022; Cole & Franke, 2017; Cumplido-Mayoral et al., 2024; Gaser et al., 2024; Kaufmann et al., 2019; Lee et al., 2022; Mishra et al., 2023; Wang et al., 2019; Wood et al., 2022; Wrigglesworth et al., 2021).

Here, we test whether brain-age estimates from ULF MRI are valid and reliable. Leveraging a unique dataset (Váša et al., 2025) in which the same participants were scanned on a HF MRI system and two identical ULF scanners, we directly assess correspondence to HF benchmarks and test-retest reliability across ULF devices. To ensure a comprehensive evaluation, we apply five diverse brain-age models and explore multiple preprocessing pipelines, including MRR and SynthSR. The resulting set of combinations provides a rich framework for systematically characterizing the potential of ULF MRI for brain-age estimation. Our broad hypothesis was that brain-age could be estimated with reasonable validity and reliability from ULF MRI, including when using brain-age models developed using HF MRI. We expected performance to vary across models, scan types, and preprocessing strategies, and therefore treated the comparison of specific pipelines as an exploratory benchmarking analysis.

## 2 Materials and Methods

### 2.1 Data

Data were from the cohort described by Váša et al. (2025). Participants (n = 23, aged 20–69 years) were recruited at King’s College London and St Thomas’s Hospital, and all provided informed consent. All participants reported being generally healthy, with no history of major neurological or psychiatric conditions. Sampling was designed to achieve approximate balance across sex and age decade (2–3 males and 2–3 females per decade). Each participant was scanned on a standard HF 3 T system (GE SIGNA Premier) and on two separate but identical ULF Hyperfine Swoop 64 mT systems (scanner software version was rc8.6.0). During the first session at King’s College London, T1-weighted (T1w) and T2-weighted (T2w) images were acquired at 1 mm³ isotropic resolution on the 3 T system, followed immediately by ULF scans using both the scanner’s standard anisotropic sequences (axial (AXI), coronal (COR), sagittal (SAG); 1.5 × 1.5 × 5 mm³ for T2w, 1.6 × 1.6 × 5 mm³ for T1w) and an additional custom isotropic (ISO; 2.2 mm³ for T2w, 2.3 mm³ for T1w) sequence. A second ULF session was conducted on a different, identical scanner at St Thomas’s Hospital within a maximum of 36 days. This paired design permitted test-retest comparisons between ULF systems and correspondence testing against the HF reference.

The scans from these three sources are referred to as HF (high-field at King’s College London), ULF1 (ultra-low-field at King’s College London) and ULF2 (ultra-low-field at St Thomas’ Hospital).

### 2.2 Multi-resolution registration (MRR)

Anisotropic ULF acquisitions were processed using MRR (Deoni et al., 2022), a form of super-resolution that allows integration of anisotropic acquisitions into isotropic space. In the present analyses, following the MRR implementation used in the original dataset paper (Váša et al., 2025), the MRR images were generated from the coronal and sagittal acquisitions only, rather than from all three orthogonal acquisitions. The resulting volumes had an approximately isotropic effective resolution of 1.5 mm³ for T2w, 1.6 mm³ for T1w. In addition, MRR pre-registration used each participant’s corresponding HF scan as the reference. Further comments on this MRR implementation are provided in the Limitations section.

### 2.3 SynthSR v2 (SSR)

SynthSR is a neural-network-based super-resolution tool that converts clinical scans of arbitrary orientation, contrast and resolution into synthetic 1 mm³ isotropic T1-weighted Magnetisation Prepared Rapid Gradient Echo (MPRAGE)-like images (Iglesias et al., 2023). In this study, version 2 of SynthSR was applied to ULF scans after MRR preprocessing. SynthSR was implemented using FreeSurfer v8.0 with the –lowfield flag and default parameters. All scans passed visual quality control (QC), both before and after SynthSR processing, using MRIcro.^1^ See Supplementary Section S2.1 for details.

### 2.4 Scan types

**Fig. 1.**
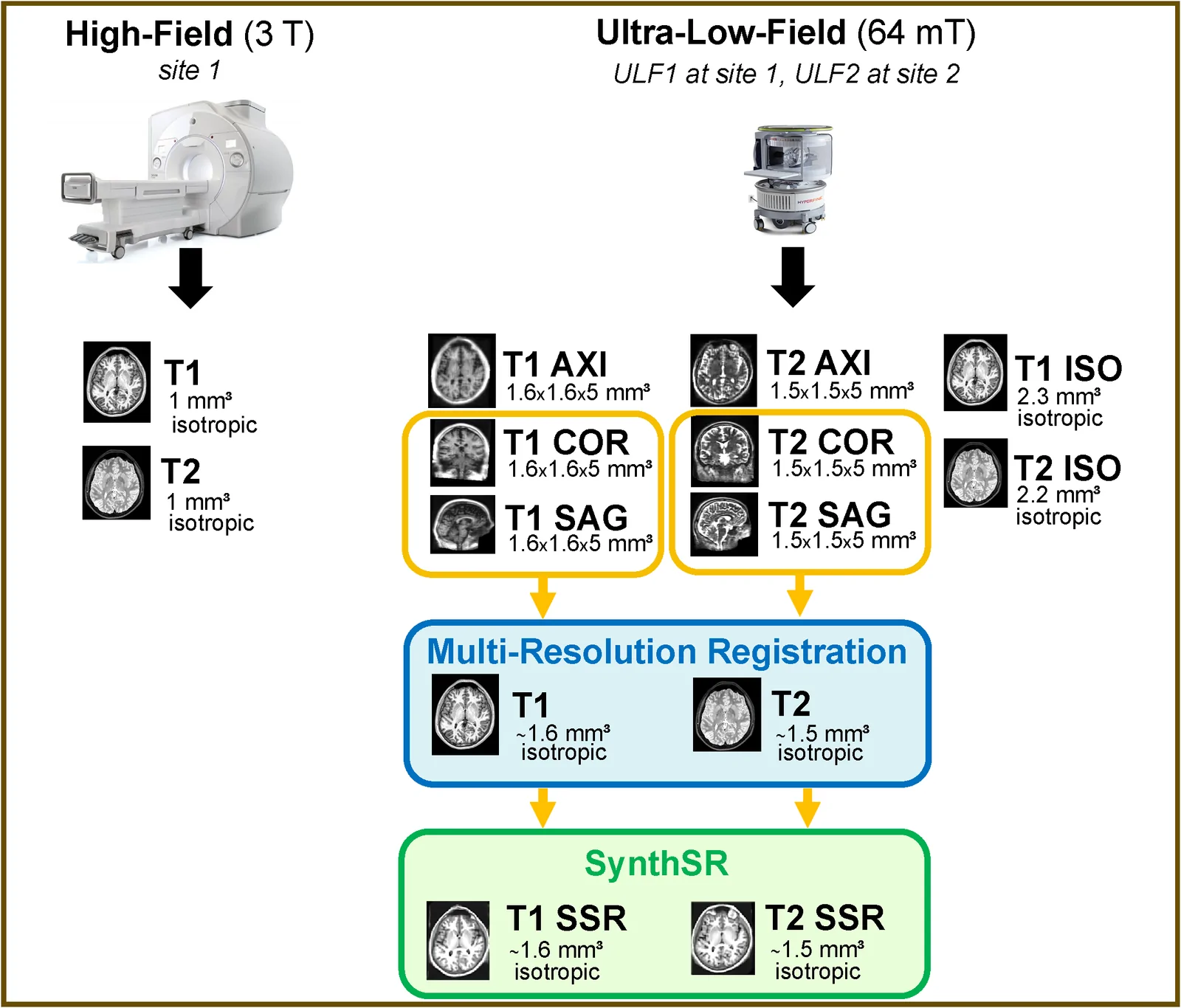
A total of 14 scan types were available. Two were acquired on the HF scanner: T1 and T2. Seven to eight single-acquisition scans were acquired on each ULF scanner: T1 and T2 in AXI, COR, SAG, and ISO orientations (with T2 ISO missing for ULF2). The COR and SAG ULF acquisitions for each scanner and contrast were then combined into an MRR volume of higher effective resolution, generating two additional scan types: T1 and T2. These MRR scans were further processed with SynthSR, resulting in two more scan types: T1 SSR and T2 SSR. For each scan type at each scanner site (HF, ULF1, ULF2), *n* = 23 participants were available, except for ULF1 T1 and its derivatives (*n* = 22). The total number of scans across scan types was *n* = 573. All five brain-age models were applied to six scan types: two HF (T1 and T2) and four ULF-derived images (T1, T2, T1 SSR, T2 SSR at both sites). The remaining eight ULF scan types were evaluated only with the best-performing brain-age model. HF, high-field; ULF1/ULF2, ultra-low-field site 1/site 2; MRR, multi-resolution registration; SSR, SynthSR; AXI, axial; SAG, sagittal; COR, coronal; ISO, isotropic.

Combining the acquired MRI data with MRR and SynthSR processing resulted in 14 distinct scan types. These are illustrated in Figure 1 (see also Supplementary Table S1) and listed here: HF scans (T1 and T2); ULF scans processed with MRR (T1 and T2); ULF scans processed with MRR and SynthSR (T1 SSR and T2 SSR); and ULF single-acquisition scans (T1 and T2 acquired in AXI, COR, SAG, and ISO orientations/resolutions). For each scan type at each scanner site (HF, ULF1, ULF2), data were available for *n* = 23 participants. Exceptions were for ULF1 T1 and its derivatives (*n* = 22), and for T2 ISO scans, which were not acquired at ULF2.

Given the large number of scans (a total of *n* = 573), the main analyses evaluating five brain-age models, focused on HF, ULF+MRR, and ULF+MRR+SSR scans (six scan types, *n* = 228 in total). The remaining eight scan types, ULF single acquisition and ISO, scan types were evaluated only with the best-performing brain-age model.

### 2.5 Brain-age models

Five different brain-age models were used to generate age predictions for each scan. Each model has its own preprocessing pipeline, typically involving image registration and/or tissue segmentation. After preprocessing, the scan quality was assessed using a QC procedure specific to each model. The deep-learning models (SynthBA, DeepBrainNet, MIDI) had the common requirement for input scans to be skull-stripped and linearly registered to a standard template. To streamline this, SynthBA’s preprocessing step was used for all three deep-learning models. The preprocessing and QC procedure for each model is described below and Table 1 summarizes which model was applied to which scan.

**Table 1.** Brain-age models (rows) applied to which scan types (columns).

	HF	HF	ULF	ULF	ULF	ULF	HF	ULF
Model	T1	T2	T1	T2	T1_SSR	T2_SSR	T1 + T2	T1 + T2
BrainageR	Yes	n/a	Yes	n/a	Yes	Yes^†^	n/a	n/a
SynthBA	Yes	Yes	Yes	Yes	Yes	Yes	n/a	n/a
MIDI-T1	Yes	n/a	Yes	n/a	Yes	Yes	n/a	n/a
MIDI-T2	n/a	Yes	n/a	Yes	n/a	n/a	n/a	n/a
DeepBrainNet	Yes	n/a	Yes	n/a	Yes	Yes^†^	n/a	n/a
PyBrainAge + FS v7.4 ra	Yes	n/a	Yes	n/a	Yes	Yes	Yes	Yes
PyBrainAge + FS v7.4 rac	Yes	Yes	Yes	Yes	No	No	n/a	n/a
PyBrainAge + FS v8.0 ra	Yes	n/a	Yes	n/a	No	No	Yes	Yes
PyBrainAge + FS v8.0 rac	Yes	Yes	Yes	Yes	Yes	Yes	n/a	n/a

All ULF scans listed here were pre-processed with MRR; “T1_SSR/T2_SSR” indicates additional SynthSR processing. The combined-input columns “HF/ULF T1 + T2” denote FreeSurfer recon-all (ra) runs that used a T1 with an accompanying T2 via the -T2 and -T2pial flags and are only applicable to the PyBrainAge model; Single-acquisition ULF (AXI, COR, SAG, ISO) scans are omitted in this table because they were evaluated only with the best-performing model (see Results).

† BrainageR and DeepBrainNet accept T1-weighted and SynthSR-derived (T1-like) inputs; here T2_SSR is treated as T1-like; ra = *recon-all*; rac = *recon-all-clinical*.

#### 2.5.1 BrainageR

BrainageR^2^ is a Gaussian Process Regression model trained on T1-weighted scans from 3,377 healthy adults (18–92 years) drawn from public datasets.^3^ The model operates on principal components derived from voxel-wise grey-matter, white-matter and CSF (cerebrospinal fluid) tissue-probability maps.

BrainageR first performs tissue segmentation and spatial normalization to MNI152 space using SPM12, after which the pretrained model is applied to generate brain-age estimates. As BrainageR was trained on T1-weighted MRI scans, we tested it only on T1-weighted, and SynthSR-derived images (which are T1-like). The quality of the preprocessing was assessed visually for each scan using the slice screenshots included in the BrainageR output, and all scans passed QC.

#### 2.5.2 SynthBA

SynthBA^4^ (Puglisi et al., 2024) is a brain-age model built on a 3D DenseNet and trained on synthetic MRIs generated from brain-tissue segmentations via a synthetic scan generator (Gopinath et al., 2024) with extensive domain randomization. Each synthetic scan is produced with randomized contrast, resolution, noise, bias fields, and intensity profiles. This enables the model to learn contrast- and resolution-agnostic features and generalise across acquisition protocols without retraining. The training segmentations were sourced from 4,105 healthy subjects, and Gaussian priors for the generator were estimated from 7,060 T1-weighted and 1,689 T2-weighted MRIs drawn from public datasets. Preprocessing involved skull-stripping using SynthStrip (Hoopes et al., 2022) followed by linear registration (rigid and affine registration) to the MNI152 template with Advanced Normalization Tools (ANTs; Tustison et al., 2021). Preprocessed scans underwent quantitative and visual QC (see Supplementary Section S2.4 for details). All scans passed QC and were used as direct inputs to SynthBA, DeepBrainNet, and MIDI. In this study, SynthBA was run using the -m g flag and tested on T1-weighted, T2-weighted, and SynthSR-derived volumes.

#### 2.5.3 DeepBrainNet (DBN)

DBN^5^ is a 2D convolutional model based on an Inception-ResNet-v2 backbone. It was trained on 11,729 T1-weighted scans from a large, multi-study lifespan cohort (ages 3–95 years) with minimal preprocessing (skull-stripping and affine registration) (Bashyam et al., 2020). Each scan is represented by 80 axial slices, and slice-level predictions are aggregated (median) to provide a scan-level brain-age estimate.

In this study, DBN was tested on T1-weighted and SynthSR-derived volumes.

#### 2.5.4 MIDI

Two pretrained models were used from the MIDI BrainAge release^6^ (Wood et al., 2022). First, the *T2 model*, (3D DenseNet-121) trained on 23,302 axial T2-weighted scans from two UK hospital networks (ages 18–95) with minimal preprocessing (DICOM-to-NIfTI, 1 mm resampling, cropping/padding, *z*-score normalization; no registration or skull-stripping). Second, the *T1 model*, (3D DenseNet-121) trained on 1,551 volumetric T1-weighted scans from a UK-based research cohort. We used the -ensemble option, which aggregates predictions across multiple random-seed checkpoints of the same T1 model to produce a single estimate.

In this study, the MIDI-T2 model was tested on T2-weighted inputs, and the MIDI-T1 ensemble was applied to T1-weighted and SynthSR-derived volumes. Because input scans were already skull-stripped and linearly registered, we used the MIDI “skull-stripped” settings, and omitted the default preprocessing steps (skull-stripping and registration to MNI152 using ANTs).

#### 2.5.5 PyBrainAge

PyBrainAge^7^ represents a more classical, feature-based approach, leveraging a well-established neuroimaging pipeline by using ridge regression on morphometric features extracted with FreeSurfer^8^ (Fischl, 2012). These include cortical thickness, surface area, and volumetric features from the Desikan-Killiany atlas. PyBrainAge was trained on 29,175 T1-weighted scans from healthy participants aged 2–100 years, across 76 different sites (mostly UK Biobank data) (Rutherford et al., 2022a).

For PyBrainAge, preprocessing involved generating the FreeSurfer-derived morphometric features used as model inputs. We varied this preprocessing step to produce different versions of these inputs, according to three factors: (i) FreeSurfer version (either v7.4.0 or v8.0.0 beta), (ii) FreeSurfer pipeline (recon-all vs recon-all-clinical), and (iii) input scan type. Not all possible combinations of these factors were exhausted: preliminary tests suggested that recon-all-clinical behaved similarly across v7.4.0 and v8.0.0, and performed better than recon-all on ULF data, so those options were prioritized. The following paragraphs describe these preprocessing combinations and relevant technical details.

*In FreeSurfer v7.4.0.* recon-all was tested on T1-weighted and SynthSR-derived scans as well as pairs of T1-weighted and T2-weighted scans using the -T2 and -T2pial flags. The command recon-all-clinical was run on T1-weighted and T2-weighted scans without SynthSR.

*In FreeSurfer v8.0.0.* recon-all was tested on T1-weighted scans without SynthSR (HF T1, ULF T1) as well as T1-weighted paired with T2-weighted using the -T2 and -T2pial flags. The command recon-all-clinical was tested on T1-weighted, T2-weighted and SynthSR-derived volumes.

*A note on the FreeSurfer variations.* The conventional recon-all command in FreeSurfer is an iterative, surface-based workflow optimized for high-resolution T1-weighted research scans (Fischl, 2012). By contrast, recon-all-clinical (introduced in v7.4) was specifically developed to extend surface reconstruction and cortical thickness estimation to heterogeneous clinical scans (e.g., T1, T2, Fluid-Attenuated Inversion Recovery - FLAIR) acquired at variable resolutions (Gopinath et al., 2025). Its key innovation is the use of domain randomisation during training. Specifically, a 3D U-Net is trained to predict isotropic signed-distance functions (SDFs) from highly augmented synthetic data that spans a wide range of contrasts, slice thicknesses, noise levels, and orientations. This enables the model to generalise to “in-the-wild” clinical data without retraining. The architecture combines this learning-based SDF prediction (referred to as “SynthDist”^9^) with FreeSurfer’s classical geometry processing to enforce topology and generate standard surfaces. In parallel, SynthSeg (Billot et al., 2023a, 2023b) provides robust volumetric labelling across contrasts and resolutions, while SynthSR is used only for visualisation and not for downstream processing (Gopinath et al., 2025).

*Harmonising FreeSurfer outputs for PyBrainAge.* The label sets from recon-all and recon-all-clinical are not identical; some structures are absent in the latter, and some common labels (notably the region denoted “CSF”) differ in neuroanatomical extent. Because PyBrainAge expects inputs derived from v7-like recon-all segmentations, a small set of targets (right/left vessel, right/left choroid plexus, and CSF) were predicted using a compact predictive model trained only on recon-all (v7.4) volumetric segmentations from the high-field data in this study. Any remaining sporadic missingness was handled with k-nearest neighbours imputation. See Supplementary Section S3 for details.

*Quality control of FreeSurfer outputs.* Quantitative and visual checks were combined. For recon-all, the free FreeSurfer Quality Control (FSQC) tool^10^ was used, along with the examination of FreeSurfer statistics (e.g., the number of surface holes per hemisphere). At the time of analyses, FSQC was not compatible with recon-all-clinical, hence for these cases, visual inspection was run using an in-house custom tool, whilst the quantitative check was run using the SynthSeg QC metrics included in the output, where a CNN-based regressor outputs scores (0–1) approximating Dice values for key brain structures, enabling unreliable segmentations to be flagged without manual ground truth (Billot et al., 2023a, 2023b). See Supplementary Section S2.3 for details.

The QC results were mixed. The T1 + T2 recon-all pipeline performed poorly overall, with 5 scans (v7.4) and 4 scans (v8.0) failing QC and the remaining ULF scans being borderline failures. For recon-all applied to SynthSR-derived T2 volumes 13 scans failed QC. Native ULF recon-all runs often appeared suboptimal on visual inspection, but none failed QC. Retaining potentially lower-quality scans was a deliberate choice, reflecting the expected lower resolution of the ULF scans. See Supplementary Section S2.2 for details. No other scan failed QC.

### 2.6 Evaluation metrics

Each brain-age model combination was assessed in three ways: (i) validity, (ii) HF-ULF correspondence and (iii) test-retest reliability.

Validity was quantified as the agreement between predicted brain-age and actual (chronological) age. Following recommendations for predictive modelling in neuroimaging (Scheinost et al., 2019), this included the predictive *R*^2^ (computed directly from predicted vs. observed values, as implemented in scikit-learn (Pedregosa et al., 2011)), alongside Pearson’s correlation (*r*) and mean absolute error (MAE).

Correspondence was quantified as the agreement between brain-age predictions from HF and ULF scans, and test-retest reliability was quantified as agreement between the two ULF scanners, both using the intraclass correlation coefficient (ICC) and the absolute symmetric percent difference (ASPD).

ICC was computed using a two-way mixed-effects model for absolute agreement and reported with 95% confidence intervals. The ASPD, as used in Sorby-Adams et al. (2024), quantifies the percentage difference between two paired measurements *X* and *Y*:

$$\text{ASPD}=100\times \frac{\left|X-Y\right|}{\left(X+Y\right)/2}$$

Higher ICC and lower ASPD both indicate better agreement. ICC summarises agreement and consistency while accounting for between-participant variability, whereas ASPD directly measures the magnitude of pairwise differences and is less influenced by the between-participant variance.

To visually assess the agreement between brain-age predictions from different scan types, Bland-Altman plots were used. For each pairing, the difference against the pairwise mean was plotted to visualise bias (mean difference) and the 95% limits of agreement (mean ± 1.96 SD).

In the main text, correspondence is reported for HF versus ULF1 only, since this was acquired on the same day at the same site and thus provide the most direct comparison. Complete results, including HF versus ULF2, are provided in Supplementary Section S4.

### 2.7 Model performance composite rankings

There were a total of 40 unique combinations of brain-age model, preprocessing pipeline, scanner and scan type. Of these 40, 26 pertained to ULF, which are the combinations of interest and are referred to as test runs. To summarize performance across metrics and enable comparison between test runs, each performance metric was ranked separately within validity, correspondence, and test-retest reliability. Ranks for MAE and ASPD were inverted to keep directions consistent. For each test run, the median rank across metrics was calculated thus obtaining a single validity ranking (brain-age vs. actual age), correspondence ranking (HF vs. ULF1), and test-retest reliability ranking (ULF1 vs. ULF2).

Due to space constraints, we show validity, correspondence, and reliability plots only for the top four performing models, selected based on validity rankings. Validity rankings were prioritised over correspondence and reliability rankings because validity reflects proximity to the ground truth (i.e., chronological age). The complete set of plots for all models is available in Supplementary Section S5.

#### 2.7.1 Application of the “best-performing model” to single-acquisition ULF scans

The best-performing model was next applied to the ULF single-acquisition sequences - the anisotropic scans (AXI, COR, SAG) and the isotropic ISO sequence - which had not been tested with any brain-age model in the main analyses. These scans are faster to acquire but lower in effective resolution than the MRR reconstructions. The preprocessing protocol for the best-performing model (see Methods section 2) was applied to these single-acquisition scans, all of which passed QC.

## 3 Results

Tables 2 and 3 present performance metrics for all 40 combinations of brain-age model, preprocessing, scanner, and scan type. Out of these 40, the key 26 test runs (ULF data only) display rankings alongside the raw metrics in the same tables. ULF2 correspondence metrics are not included in Tables 2 and 3, but can be found in a larger table in Supplementary Section S4.

**Table 2.** Validity.

Test	Model	Scanner	Scan type	failed *n*	*r*	*R* ^2^	MAE	Validity ranking
	*BrainageR*	*HF*	*T1*	*0*	*0.91*	*0.81*	*4.99*	
1	BrainageR	ULF1	T1	2	0.40	0.03	11.77	24
	BrainageR	ULF2	T1	4	-0.12	-0.39	13.72	
2	BrainageR	ULF1	T1_SSR	0	0.81	0.64	7.52	4
	BrainageR	ULF2	T1_SSR	0	0.78	0.57	8.14	
3	BrainageR	ULF1	T2_SSR	0	0.77	0.53	8.24	5
	BrainageR	ULF2	T2_SSR	0	0.82	0.60	7.29	
	*SynthBA*	*HF*	*T1*	*0*	*0.89*	*0.52*	*8.23*	
4	SynthBA	ULF1	T1	0	0.76	0.02	12.94	18
	SynthBA	ULF2	T1	0	0.76	0.08	11.92	
	*SynthBA*	*HF*	*T2*	*0*	*0.80*	*0.55*	*7.40*	
5	SynthBA	ULF1	T2	0	0.83	0.64	6.94	1
	SynthBA	ULF2	T2	0	0.81	0.61	6.70	
6	SynthBA	ULF1	T1_SSR	0	0.84	0.52	8.21	10
	SynthBA	ULF2	T1_SSR	0	0.70	0.22	10.86	
7	SynthBA	ULF1	T2_SSR	0	0.89	0.29	9.87	13
	SynthBA	ULF2	T2_SSR	0	0.88	0.13	11.52	
	*MIDI-T1*	*HF*	*T1*	*0*	*0.96*	*0.18*	*12.02*	
8	MIDI-T1	ULF1	T1	0	0.70	0.19	9.93	16
	MIDI-T1	ULF2	T1	0	0.57	0.03	11.37	
	*MIDI-T2*	*HF*	*T2*	*0*	*0.95*	*0.79*	*5.67*	
9	MIDI-T2	ULF1	T2	0	0.88	0.54	8.21	2
	MIDI-T2	ULF2	T2	0	0.92	0.60	7.59	
10	MIDI-T1	ULF1	T1_SSR	0	0.60	-0.20	13.22	21
	MIDI-T1	ULF2	T1_SSR	0	0.78	-0.20	13.32	
11	MIDI-T1	ULF1	T2_SSR	0	0.82	-0.25	13.81	23
	MIDI-T1	ULF2	T2_SSR	0	0.86	-0.30	14.20	
	*DeepBrainNet*	*HF*	*T1*	*0*	*0.96*	*0.73*	*6.49*	
12	DeepBrainNet	ULF1	T1	0	0.52	-1.26	19.27	26
	DeepBrainNet	ULF2	T1	0	0.63	-1.07	18.25	
13	DeepBrainNet	ULF1	T1_SSR	0	-0.06	-0.50	13.83	22
	DeepBrainNet	ULF2	T1_SSR	0	0.65	-0.14	12.02	
14	DeepBrainNet	ULF1	T2_SSR	0	0.77	0.51	9.46	9
	DeepBrainNet	ULF2	T2_SSR	0	0.72	0.44	10.03	
	*Py_FSv7_ra*	*HF*	*T1*	*0*	*0.84*	*0.65*	*7.05*	
15	Py_FSv7_ra	ULF1	T1	0	0.66	-0.01	12.40	20
	Py_FSv7_ra	ULF2	T1	0	0.52	-0.17	13.20	
	*Py_FSv7_ra*	*HF*	*T1 + T2*	*0*	*0.83*	*0.67*	*6.88*	
16	Py_FSv7_ra	ULF1	T1 + T2	1	0.59	-0.75	16.94	25
	Py_FSv7_ra	ULF2	T1 + T2	4	0.63	-0.49	16.43	
17	Py_FSv7_ra	ULF1	T1_SSR	0	0.67	0.45	9.26	7
	Py_FSv7_ra	ULF2	T1_SSR	0	0.70	0.49	8.85	
18	Py_FSv7_ra	ULF1	T2_SSR	6	0.68	0.46	9.23	17
	Py_FSv7_ra	ULF2	T2_SSR	7	0.29	-0.01	11.56	
	*Py_FSv7_rac*	*HF*	*T1*	*0*	*0.75*	*0.32*	*9.40*	
19	Py_FSv7_rac	ULF1	T1	0	0.78	0.56	7.99	6
	Py_FSv7_rac	ULF2	T1	0	0.67	0.42	9.12	
	*Py_FSv7_rac*	*HF*	*T2*	*0*	*0.75*	*0.21*	*10.22*	
20	Py_FSv7_rac	ULF1	T2	0	0.71	0.32	10.19	14=
	Py_FSv7_rac	ULF2	T2	0	0.69	0.31	9.90	
	*Py_FSv8_ra*	*HF*	*T1*	*0*	*0.80*	*0.36*	*9.79*	
21	Py_FSv8_ra	ULF1	T1	1	0.71	0.29	10.11	14=
	Py_FSv8_ra	ULF2	T1	1	0.73	0.29	9.47	
	*Py_FSv8_ra*	*HF*	*T1 + T2*	*0*	*0.77*	*0.35*	*9.38*	
22	Py_FSv8_ra	ULF1	T1 + T2	1	0.63	-0.33	14.12	19
	Py_FSv8_ra	ULF2	T1 + T2	3	0.78	0.06	11.76	
	*Py_FSv8_rac*	*HF*	*T1*	*0*	*0.74*	*0.32*	*9.47*	
23	Py_FSv8_rac	ULF1	T1	0	0.77	0.54	8.25	8
	Py_FSv8_rac	ULF2	T1	0	0.67	0.41	9.43	
24	Py_FSv8_rac	ULF1	T1_SSR	0	0.77	0.59	7.37	3
	Py_FSv8_rac	ULF2	T1_SSR	0	0.76	0.57	8.06	
	*Py_FSv8_rac*	*HF*	*T2*	*0*	*0.75*	*0.17*	*10.42*	
25	Py_FSv8_rac	ULF1	T2	0	0.73	0.33	10.17	12
	Py_FSv8_rac	ULF2	T2	0	0.72	0.35	9.86	
26	Py_FSv8_rac	ULF1	T2_SSR	0	0.71	0.32	9.88	11
	Py_FSv8_rac	ULF2	T2_SSR	0	0.71	0.35	9.29	

All conditions start with *n* = 23, except for ULF1 T1 derived conditions (*n* = 22). The number of scans removed due to failed preprocessing/QC is shown in “failed *n*”. The validity ranking is based on the median of six per-metric ranks within each “Test“ run (i.e., the three performance metrics, each evaluated for ULF1 and ULF2, are first converted to ranks, and the median of those six ranks is then used for the final ranking). Numerically-low ranks indicate better performance whilst numerically-high ranks indicate poor performance; HF: high-field; ULF1/ULF2: ultra-low-field at site 1/site 2; SSR: SynthSR; *r*: Pearson’s correlation coefficient; *R*^2^: coefficient of determination; MAE: mean absolute error; “=” denotes tied ranks; Py_FSv7_ra: PyBrainAge using Freesurfer (FS) v7.4 and (recon-all); Py_FSv8_rac: PyBrainAge using FS v8.0 and (recon-all-clinical).

**Table 3.** Correspondence and reliability.

					Correspondence						Test-retest					
Test	Model	Scan type	HF type	max failed *n*	*r*	ASPD (CI)		ICC (CI)		Ranking	*r*	ASPD (CI)		ICC (CI)		Ranking
1	BrainageR	T1	T1	6	0.37	15.3	(7.5-34.1)	0.30	(-0.15-0.65)	23	0.37	3.2	(1.1-19.7)	0.38	(-0.14-0.73)	25
2	BrainageR	T1_SSR	T1	0	0.84	16.6	(6.8-23.5)	0.77	(0.52-0.90)	7	0.97	3.3	(1.0-6.7)	0.97	(0.93-0.99)	5
3	BrainageR	T2_SSR	T1	0	0.86	17.3	(9.1-25.4)	0.77	(0.35-0.91)	8	0.97	5.0	(3.1-8.7)	0.97	(0.93-0.99)	8
4	SynthBA	T1	T1	0	0.84	16.7	(7.3-28.2)	0.69	(0.19-0.88)	9	0.98	4.0	(2.3-7.4)	0.98	(0.95-0.99)	2=
5	SynthBA	T2	T2	0	0.93	7.6	(2.4-16.4)	0.92	(0.80-0.97)	1	0.99	1.7	(1.0-3.7)	0.99	(0.99-1.00)	1
6	SynthBA	T1_SSR	T1	0	0.68	27.1	(14.7-40.7)	0.64	(0.31-0.83)	21	0.86	10.9	(5.0-25.2)	0.86	(0.69-0.94)	19
7	SynthBA	T2_SSR	T2	0	0.81	34.6	(20.0-57.5)	0.57	(-0.08-0.85)	16	0.97	8.0	(2.1-17.5)	0.97	(0.93-0.99)	8
8	MIDI-T1	T1	T1	0	0.67	24.8	(13.0-36.3)	0.47	(0.02-0.75)	18	0.82	4.9	(2.4-9.1)	0.82	(0.62-0.92)	20=
9	MIDI-T2	T2	T2	0	0.93	12.9	(5.6-22.3)	0.81	(0.60-0.91)	3=	0.98	3.7	(2.5-5.4)	0.98	(0.94-0.99)	2=
10	MIDI-T1	T1_SSR	T1	0	0.62	16.5	(5.7-28.6)	0.57	(0.20-0.80)	15	0.83	4.6	(3.1-9.0)	0.81	(0.60-0.92)	20=
11	MIDI-T1	T2_SSR	T2	0	0.87	25.9	(12.3-31.4)	0.54	(-0.03-0.81)	17	0.97	3.5	(1.7-5.9)	0.97	(0.92-0.99)	5
12	DeepBrainNet	T1	T1	0	0.54	65.4	(29.9-81.6)	0.10	(-0.07-0.38)	25	0.88	3.0	(1.9-5.3)	0.87	(0.72-0.95)	16=
13	DeepBrainNet	T1_SSR	T1	0	-0.11	23.0	(16.3-44.5)	-0.09	(-0.52-0.35)	26	-0.08	5.6	(3.3-13.1)	-0.07	(-0.44-0.35)	26
14	DeepBrainNet	T2_SSR	T1	0	0.85	23.1	(9.2-42.1)	0.57	(0.06-0.82)	14	0.96	3.9	(3.1-5.9)	0.96	(0.90-0.98)	10
15	Py_FSv7_ra	T1	T1	0	0.66	26.2	(11.9-48.0)	0.43	(-0.08-0.75)	19	0.79	6.1	(2.6-15.3)	0.79	(0.56-0.91)	22
16	Py_FSv7_ra	T1 + T2	T1	5	0.71	33.7	(21.8-55.6)	0.40	(-0.11-0.75)	22	0.89	5.1	(3.7-7.6)	0.90	(0.75-0.96)	15
17	Py_FSv7_ra	T1_SSR	T1	0	0.74	18.0	(6.9-28.3)	0.70	(0.41-0.86)	10=	0.87	9.6	(5.3-15.4)	0.88	(0.73-0.95)	18
18	Py_FSv7_ra	T2_SSR	T1	13	0.71	9.8	(4.9-32.5)	0.67	(0.30-0.86)	10=	0.62	13.1	(6.0-20.2)	0.63	(0.13-0.87)	24
19	Py_FSv7_rac	T1	T1	0	0.93	24.5	(18.7-29.0)	0.66	(-0.06-0.91)	12=	0.93	7.2	(2.9-11.2)	0.93	(0.85-0.97)	11=
20	Py_FSv7_rac	T2	T2	0	0.87	12.3	(9.1-16.1)	0.81	(0.56-0.92)	3=	0.92	5.2	(3.3-8.0)	0.92	(0.82-0.97)	13=
21	Py_FSv8_ra	T1	T1	2	0.89	28.9	(24.0-37.1)	0.42	(-0.04-0.80)	20	0.65	11.3	(6.2-14.0)	0.65	(0.32-0.84)	23
22	Py_FSv8_ra	T1 + T2	T1	4	0.73	43.4	(25.8-51.3)	0.23	(-0.05-0.62)	24	0.88	3.9	(2.3-6.6)	0.88	(0.72-0.95)	16=
23	Py_FSv8_rac	T1	T1	0	0.92	24.4	(16.2-30.5)	0.66	(-0.07-0.91)	12=	0.92	7.2	(3.0-11.2)	0.92	(0.81-0.97)	13=
24	Py_FSv8_rac	T1_SSR	T1	0	0.88	20.5	(6.3-27.0)	0.72	(-0.04-0.91)	6	0.97	5.5	(2.3-7.1)	0.97	(0.93-0.99)	8
25	Py_FSv8_rac	T2	T2	0	0.87	13.0	(7.5-17.7)	0.81	(0.50-0.92)	5	0.93	4.3	(2.2-7.4)	0.93	(0.85-0.97)	11=
26	Py_FSv8_rac	T2_SSR	T2	0	0.93	12.2	(4.2-14.4)	0.89	(0.55-0.96)	2	0.98	4.2	(1.8-5.8)	0.97	(0.94-0.99)	5

Correspondence denotes agreement between ULF1 scans and a HF homologue (see “HF type” column). Test–retest reliability denotes agreement between ULF1 and ULF2. Starting sample size is *n* = 23 except for ULF1 T1 derived scans *n* = 22. Max scans excluded due to failed preprocessing/QC shown in “max failed *n*” (refer to ULF only; all HF passed). Correspondence and test–retest rankings are based on the median of three per-metric ranks, where each of the metrics, *r*, ASPD and ICC is first converted to a rank and then the median of these three ranks is used. Numerically-low ranks indicate good performance while numerically-high ranks indicate poor performance; HF, high-field; ULF1/ULF2, ultra-low-field at site 1/site 2; SSR, SynthSR; *r*, Pearson correlation; ASPD, absolute symmetric percent difference; ICC, intraclass correlation coefficient; CI, confidence interval; “=” denotes tied ranks; Py_FSv7_ra, PyBrainAge using Freesurfer (FS) v7.4 and (recon-all); Py_FSv8_rac, PyBrainAge using FS v8.0 and (recon-all-clinical).

Performance metrics varied widely. For validity, the HF metrics (the benchmark) showed numerically better performance and tighter ranges than ULF scans (HF: r=0.74 –0.96, $R^{2}\text{​}\;=0.17$ –0.81, MAE =4.99 –12.02 years; ULF: r=−0.12 –0.92, $R^{2}\text{​}\;=-1.26$ –0.64, MAE = 6.7–19.27 years). For HF-ULF correspondence, the ranges were r=−0.11 –0.93, ASPD =7.6 –65.4, ICC =−0.09 –0.92. For test-retest reliability, ranges were r=−0.08 –0.99, ASPD =1.7 –13.1, ICC =−0.07 –0.99.

**Fig. 2.**
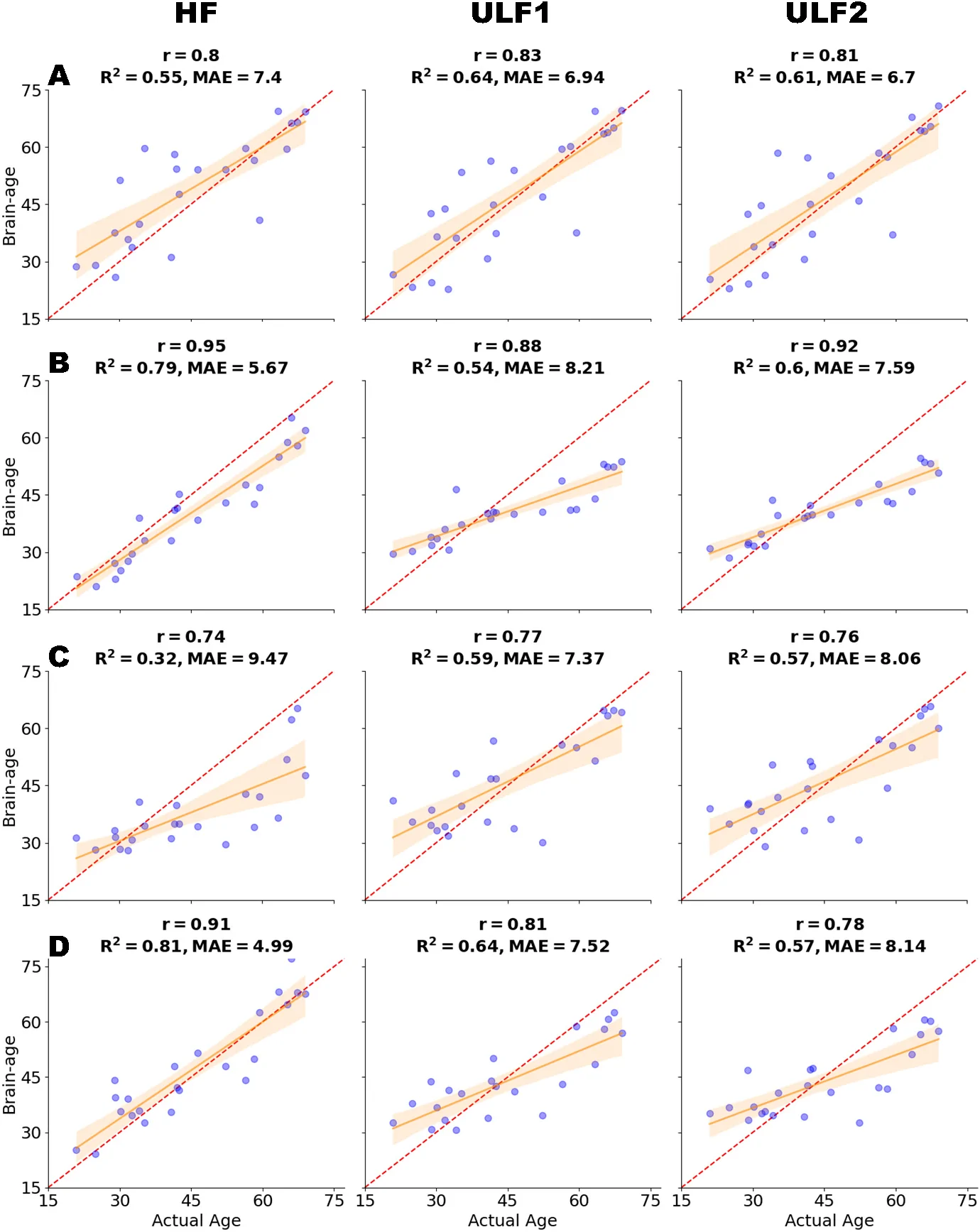
Validity plots for the top four performing brain-age models. Rows (A–D) show model/scan combinations: (A) SynthBA on T2; (B) MIDI (T2 model) on T2; (C) PyBrainAge on T1_SSR (FreeSurfer v8, recon-all-clinical); and (D) BrainageR on T1_SSR. Columns are HF (high-field), ULF1, and ULF2 (ultra-low-field sites 1/2). Each panel plots estimated brain-age versus actual (chronological) age; the red dashed diagonal is the identity line. The solid line is the fitted regression with a confidence interval. Annotations report the Pearson correlation *r*, the coefficient of determination *R*^2^, and the mean absolute error (MAE).

**Fig. 3.**
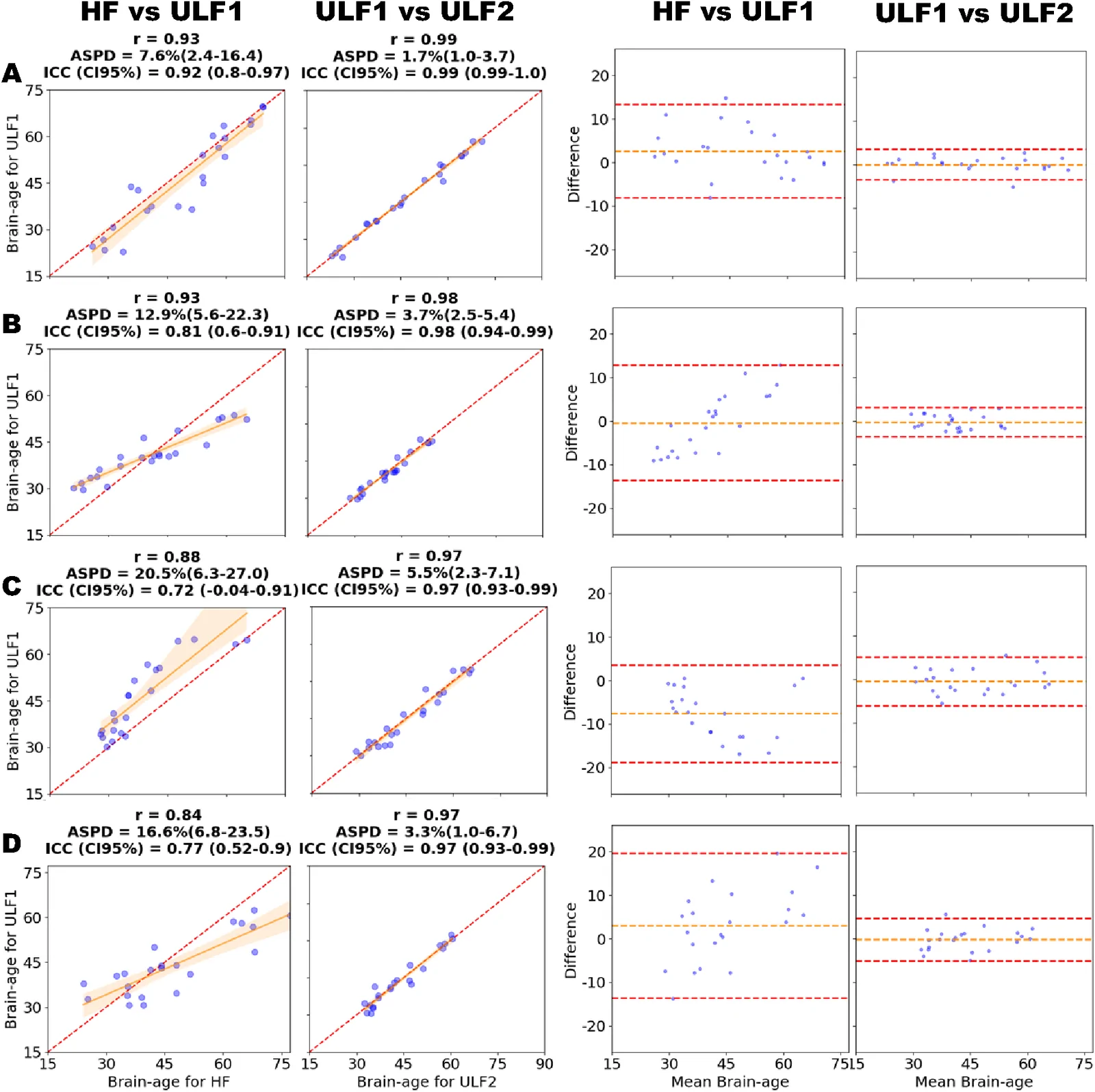
Correspondence and reliability plots for the top four performing brain-age models. Rows (A–D) show model and scan type combinations: (A) SynthBA on T2; (B) MIDI (T2 model) on T2; (C) PyBrainAge on T1_SSR (FreeSurfer v8, recon-all-clinical); and (D) BrainageR on T1_SSR. Columns are: (1) Correspondence (HF vs. ULF1), (2) test-retest reliability (ULF1 vs. ULF2), (3) Bland–Altman for HF vs. ULF1, and (4) Bland–Altman for ULF1 vs. ULF2. Correspondence and test-retest panels (columns 1–2) show scatter plots comparing estimated brain-age between scanner pairs; annotations report the Pearson correlation *r*, absolute symmetric percent difference (ASPD) with 95% confidence interval (CI), and intraclass correlation coefficient (ICC) with 95% CI. Bland–Altman panels (columns 3–4) plot the pairwise difference versus the pairwise mean; the central orange dashed line indicates the mean bias and the outer dashed red lines show the 95% limits of agreement. HF = high-field; ULF1/ULF2 = ultra-low-field sites 1/2; SSR = SynthSR.

Based on validity rankings, the top four brain-age models (in descending order) were SynthBA on T2 scans, MIDI on T2 scans, PyBrainAge using recon-all-clinical on T1 scans with SynthSR, and BrainageR on T1 scans with SynthSR. For these four combinations, validity plots are shown in Figure 2, while correspondence, test-retest reliability and Bland–Altman plots are shown in Figure 3. For a complete set of plots, see Supplementary Section S5.

In validity and correspondence plots, perfect performance would be indicated by datapoints aligning along the line of identity. For the top four model combinations, HF validity closely approached this pattern (r=0.74 –0.95, $R^{2}\text{​}\;=0.32$ –0.81, MAE =4.99 –9.47 years). ULF1 and ULF2 showed broadly similar patterns, although estimates lay somewhat further from the line of identity (r=0.76 –0.92, $R^{2}\text{​}\;=0.54$ –0.64, MAE =6.7 –8.21 years).

Correspondence showed good results, with strong performance for SynthBA (r=0.93 , ASPD =7.6% ; 95% CI: 2.4–16.4; ICC = 0.92, 95% CI: 0.80–0.97) and moderate-to-strong for the other three top-performing models (r=0.84 –0.93, ASPD =12.9 –20.5%, ICC =0.72 –0.81). However, for PyBrainAge the ICC estimate was 0.72, and the 95% CI included zero (−0.04 –0.91), indicating that agreement was not statistically significant. For SynthBA, the Bland–Altman plot did not reveal any obvious age-related bias. In contrast, the plots for MIDI and BrainageR indicated that ULF scans tended to underestimate older ages and overestimate younger ages relative to HF, while PyBrainAge showed a systematic overestimation relative to HF. Test-retest reliability for the top-performing model combinations was strong (r=0.97 –0.99, ASPD = 1.7–5.5%, ICC = 0.97–0.99). Bland–Altman plots did not reveal any noticeable age-related bias. The complete correspondence results, including HF versus ULF2, are provided in Supplementary Section S4.

The best-performing model combination based on validity rankings was SynthBA on T2 scans, which also ranked first in both correspondence and test-retest reliability rankings. As the best-performing model, SynthBA was further tested on single-acquisition scans; these results are shown in Table 4 and the plots can be found in Supplementary Section S5.2.1. Validity metrics ranged from weak to strong r=0.65 –0.83, $R^{2}\text{​}\;=-0.70$ –0.62, MAE = 6.82–16.74 years, while test-retest reliability was strong (r=0.86 –0.99, ASPD =1.7 –6.7%, ICC =0.86 –0.99).

**Table 4.** Best-performing model: SynthBA.

		Validity			Test-retest				
Scan type	Scanner	*r*	*R* ^2^	MAE	*r*	ASPD (CI)		ICC (CI)	
T1 (MRR)	ULF1	0.76	0.02	12.94	0.98	4.0	(2.3-7.4)	0.98	(0.95-0.99)
T1 (MRR)	ULF2	0.76	0.08	11.92					
T2 (MRR)	ULF1	0.83	0.64	6.94	0.99	1.7	(1.0-3.7)	0.99	(0.99-1.00)
T2 (MRR)	ULF2	0.81	0.61	6.70					
T1_AXI	ULF1	0.71	0.04	10.48	0.95	5.2	(3.1-8.2)	0.95	(0.88-0.98)
T1_AXI	ULF2	0.69	0.03	11.10					
T1_SAG	ULF1	0.78	0.39	9.99	0.97	6.7	(3.5-12.8)	0.96	(0.91-0.98)
T1_SAG	ULF2	0.78	0.45	8.62					
T1_COR	ULF1	0.74	-0.05	12.45	0.98	5.0	(2.4-10.6)	0.98	(0.96-0.99)
T1_COR	ULF2	0.75	-0.05	12.39					
T1_ISO	ULF1	0.65	-0.70	16.74	0.86	6.5	(2.1-12.2)	0.86	(0.71-0.94)
T1_ISO	ULF2	0.76	-0.52	15.71					
T2_AXI	ULF1	0.78	0.41	8.97	0.98	2.3	(1.2-4.8)	0.98	(0.94-0.99)
T2_AXI	ULF2	0.78	0.42	8.68					
T2_SAG	ULF1	0.72	0.08	11.80	0.97	1.7	(1.0-3.9)	0.97	(0.93-0.99)
T2_SAG	ULF2	0.78	0.22	10.53					
T2_COR	ULF1	0.81	0.57	7.25	0.99	3.0	(1.0-4.5)	0.99	(0.97-1.00)
T2_COR	ULF2	0.83	0.62	6.82					
T2_ISO	ULF1	0.74	0.44	8.84					

The first four rows recapitulate the validity and test-retest reliability results for SynthBA using T1 and T2 scans (MRR, without SSR), for reference. The remaining rows are hitherto unreported results: the performance metrics for the single acquisition ULF scans (no MRR/SSR) using SynthBA. HF, high-field; ULF1/ULF2, ultra-low-field at site 1/site 2; SSR, SynthSR; *r*, Pearson correlation; *R*^2^: coefficient of determination; MAE: mean absolute error; ASPD, absolute symmetric percent difference; ICC, intraclass correlation coefficient; CI, confidence interval.

### 3.1 A closer look at PyBrainAge and the impact of FreeSurfer variations

Within the PyBrainAge iterations, three additional findings were observed. First, the FreeSurfer software version (v7.4.0 vs. v8.0.0) appeared to influence results. Using the standard recon-all command, v8.0.0 resulted in higher validity rankings than v7.4.0 (e.g., with T1 scans in ULF1, $R^{2}\text{​}\;=-0.01$ [rank 20] vs. $R^{2}\text{​}\;=0.29$ [rank 14]). By contrast, version differences seemed to have less impact when using the recon-all-clinical command, which was introduced in v7.4.0 (e.g., $R^{2}\text{​}\;=0.56$ [rank 6] in v7.4.0 vs. $R^{2}\text{​}\;=0.54$ [rank 8] in v8.0.0). Second, ULF brain-age estimation improved when using recon-all-clinical instead of recon-all, or when using SynthSR instead of no treatment. For example, PyBrainAge with recon-all and T1 in ULF1 achieved $R^{2}\text{​}\;=-0.01$ (rank 20), improving to $R^{2}\text{​}\;=0.56$ (rank 6) with recon-all-clinical and $R^{2}\text{​}\;=0.45$ (rank 7) with SynthSR. The best performance was achieved when both were applied in combination ($R^{2}\text{​}\;=0.59$ , rank 3, v8.0.0). Third, adding T2 to aid T1 segmentation (using the -T2 and -T2pial flags) unexpectedly reduced performance (e.g., rank 20 dropped to rank 25 in v7.4.0; rank 14 dropped to rank 19 in v8.0.0).

## 4 Discussion

In this study, we evaluated the validity, correspondence, and test-retest reliability of brain-age estimates derived from ULF MRI using five diverse brain-age models and multiple preprocessing pipelines. This design resulted in an extensive set of combinations, allowing us to benchmark performance systematically.

Our key finding is that ULF brain-age estimates derived from T1 or T2 scans can achieve high validity and reliability, comparable to HF benchmarks. To illustrate this, we highlight the top four performing model combinations, which capture broader patterns observed across pipelines. These were selected based on validity rankings and consisted of (in descending rank order): SynthBA on T2 scans, MIDI on T2 scans, PyBrainAge using recon-all-clinical on T1 scans with SynthSR, and BrainageR on T1 scans with SynthSR.

This convergence across the three evaluation axes is notable. Validity results showed that ULF estimates closely tracked actual (chronological) age (with *r* and *R*^2^ values ranging from 0.77 to 0.88 and 0.54 to 0.64, respectively). Correspondence results demonstrated strong agreement with HF estimates (ICC ranging from 0.72 to 0.92). Test-retest reliability results confirmed high reproducibility across two ULF scanners (ICC ranging from 0.97 to 0.99). Together, these findings support the robustness of repeated acquisitions in ULF for this scanner and are consistent with prior reports using the same dataset (Váša et al., 2025). These results therefore highlight the potential for ULF-derived brain-age estimates to be both accurate and reliable, a critical prerequisite for use as a biomarker in research and clinical settings.

Beyond their strong performance, findings from the top four combinations revealed other patterns. The top models were based on a range of different input data types: raw scans (MIDI, SynthBA), voxel-wise tissue volumes (BrainageR), or morphometric parcellations (PyBrainAge, which uses FreeSurfer segmentations). This suggests that robust features important for brain-age estimation in ULF can be extracted at both fine and coarse spatial scales. This interpretation aligns with previous ULF fidelity studies reporting moderately strong HF-to-ULF correspondence for both coarse parcellations (Váša et al., 2025) and fine-grained vertex-level structures (Pretzsch et al., 2025). Another systematic feature was a consistent negative bias relative to the identity line (Fig. 2), reflected in shallower regression slopes and consistent with the known regression-to-the-mean effect (de Lange & Cole, 2020), whereby predictions are biased toward the mean age of the training sample.

A further important finding was that brain-age performance depended strongly on the precise combination of preprocessing, brain-age model and scan type. This is consistent with recent evidence also showing that the accuracy of ULF-derived anatomical features depends on the processing used (Pretzsch et al., 2025). For example, applying SynthSR to T1 scans improved validity ranks from 24th to 4th in BrainageR and from 20th to 7th in PyBrainAge v7.4.0 with recon-all. However, in some cases the gains were minimal and in others even detrimental. For example, the two highest validity rankings were SynthBA and MIDI on T2 scans without SynthSR, where their treatment with SynthSR actually reduced performance (ranks reduced from 1st to 13th and 2nd to 23rd, respectively). In addition, in SynthBA, some anisotropic acquisitions performed closely to the MRR-processed isotropic scans (constructed from coronal and sagittal anisotropic acquisitions; e.g., T2 (MRR): r=0.81 , $R^{2}\text{​}\;=0.61$ , MAE =6.70 vs. T2_COR: r=0.83 , $R^{2}\text{​}\;=0.62$ , MAE = 6.82), further indicating that MRR processing, another form of super-resolution, may not be essential for certain brain-age models and that these findings are unlikely to depend solely on the specific MRR parameters used here. By contrast, PyBrainAge benefited from extensive image enhancement and performed best when combining MRR, SSR, and the recon-all-clinical pipeline (incorporating SynthSeg), rather than the classical recon-all command. Scan modality also made a difference, whereby each model showed distinct strengths and weaknesses which could not always be readily explained by the modality native to its training. For instance, SynthBA (training included both T1 and T2 scans) and MIDI (trained separately for T1 and T2) excelled with T2 but were weaker with T1 (validity ranks reduced from 1st to 18th and 2nd to 16th, respectively). In contrast, a strong and balanced performance across T1 and T2 modalities was observed for BrainageR once SynthSR was applied (validity rankings of 4th and 5th for T1_SSR and T2_SSR, respectively). SynthSR effectively converts a T2 scan to a T1 scan, matching the BrainageR training data.

Taken together, these findings point to three broader patterns. First, the results suggest that models trained on HF datasets with greater variability in scan quality (e.g., including noisy, lower-quality or synthetic scans, as in MIDI and SynthBA) can perform robustly even without SynthSR. By contrast, models trained on datasets that are diverse in site or study but still composed mainly of research-grade scans (e.g., BrainageR, PyBrainAge, DBN) appear more reliant on SynthSR for adequate performance with ULF data. However, confirming this trend would require a dedicated investigation, which was beyond the scope of the present study. Second, they suggest that image enhancement techniques like SynthSR warrant careful consideration, as different models may exhibit varying sensitivities, and additional processing can sometimes yield diminishing or even negative returns. Third, they further suggest that robust brain-age estimates are attainable even without acquisition of multiple orthogonal anisotropic scans, and subsequent preprocessing using MRR, as observed with certain single-acquisition results. Clinically, this may be advantageous, as sparse acquisitions in orthogonal planes are often faster to inspect (as cited in Billot et al., 2023b). Beyond workflow, multiple shorter anisotropic acquisitions may also reduce the risk of motion artefacts compared with a single longer high-resolution scan (Baljer et al., 2025a, 2025b), an advantage that may be important in populations for whom lying still is challenging, such as children (Scheinost et al., 2019). Furthermore, our results suggest that even within single-slice acquisitions, some orientations may be preferable: with SynthBA, sagittal slices performed best for T1 ($R^{2}\text{​}\;=0.45$ vs. $R^{2}\text{​}\;=0.03$ axial, $R^{2}\text{​}\;=-0.05$ coronal in ULF2), while coronal slices performed best for T2 ($R^{2}\text{​}\;=0.62$ vs. $R^{2}\text{​}\;=0.42$ axial, $R^{2}\text{​}\;=0.22$ sagittal in ULF2). Although additional benchmarking is needed, such orientation-specific differences could help guide protocol design when only limited acquisitions are feasible.

Consistent with this broader pattern, an in-depth look at findings from PyBrainAge—which relies on FreeSurfer-derived morphometric features—showed that the choice of preprocessing strongly influenced outcomes. For example, both the FreeSurfer version (v7.4 vs v8.0) and command type (recon-all vs. recon-all-clinical) affected brain-age performance. Such variability is consistent with previous reports of segmentation differences across FreeSurfer versions (Whelan et al., 2016) and further underscores how preprocessing choices can shape ULF brain-age estimation, an effect that has also been observed in other imaging studies (Botvinik-Nezer et al., 2020; Haller et al., 2016; Pretzsch et al., 2025; Sederevičius et al., 2021). It is also worth bearing in mind that FreeSurfer-based processing substantially increases computational time compared with the other brain-age models tested here, which is an important practical consideration for future applications.

### 4.1 Limitations and future work

This study has several limitations. First, the sample size was modest, so chance findings, for example in the form of unstable pipeline rankings and their differences, cannot be excluded. This is particularly relevant for validity metrics such as MAE and *R*^2^, which can be unstable in small samples, hence these values should be interpreted cautiously. However, even though the modest sample size limits precision, the repeated-measures design ensured that all comparisons were made within individuals, reducing variability. This increases confidence that observed differences across pipelines reflect genuine methodological sensitivities rather than noise, especially as validity was assessed against chronological age (the ground truth) and correspondence to HF benchmarks. In addition, we reported confidence intervals for ICCs in the correspondence and test-retest reliability analyses, which often excluded zero and therefore supported the presence of a reliable signal, particularly for ULF test-retest reliability. On a related note, the comparisons described in the discussion are based on rankings rather than formal statistical tests. This reflects the exploratory nature of this study, where our aim was not to test specific hypotheses, but rather to assess the general feasibility of brain-age estimation in ULF via systematic evaluation of a landscape of possible model-pipeline-scan combinations.

Second, the possibility that image-enhancement methods introduce spurious anatomical features (hallucinations), cannot be excluded; this has been noted as a general concern for super-resolution approaches (Lin et al., 2023), and may also apply to SynthSR. Notably, SynthSR is explicitly designed to inpaint lesions (including white matter hyperintensities), which could pose problems when applied in older individuals or patient populations. However, the strong performance of pipelines that do not rely on SynthSR (e.g., MIDI with T2) suggests this is unlikely to be the sole explanation of our robust ULF brain-age findings. Nevertheless, emerging approaches such as SuperSynth^11^ now offer super-resolution without lesion inpainting.

A third limitation concerns generalizability. The sample, was modestly sized and non-randomly selected, which raises the possibility of bias in the cohort. In addition, although we refer to the scanners as HF and ULF, the results may not extend to other scanner models or field strengths or acquisition parameters (Pretzsch et al., 2025). Finally, while we tested five established brain-age models across a rich combination of pipelines, our analyses were not exhaustive and other widely used frameworks were not included.

A further limitation concerns the MRR implementation used in the present analyses. Although MRR was originally intended to combine three orthogonal anisotropic acquisitions, the implementation used here generated MRR images from the coronal and sagittal acquisitions only, as per dataset paper (Váša et al., 2025). In addition, MRR pre-registration used each participant’s corresponding HF scan as the reference, which limits direct applicability to datasets without matched HF scans. A correction to the original dataset paper (Váša et al., 2026) has since evaluated both the inclusion of the axial acquisition and the replacement of HF-based pre-registration with pre-registration to a standard MNI template. These analyses indicated only minor effects on regional morphometric outputs, and the corrected results remained consistent with the original conclusions of the dataset paper. The present brain-age analyses were not re-run using the corrected MRR images, so the impact of these MRR implementation choices on brain-age estimates remains unknown. However, given the minor impact observed for regional morphometric outputs, and the comparable performance of some single-orientation anisotropic scans (without MRR) reported above, a substantial impact on the present brain-age results seems unlikely. Moreover, with respect to the number of MRR inputs, including the axial acquisition would be expected, if anything, to improve the reconstruction or leave it broadly unchanged, rather than reduce performance.

These considerations point to aims for future work. A key step will be to extend this work by testing whether ULF-derived brain-age estimates can serve as predictors of health outcomes, thereby establishing their translational validity. In addition, replication in larger samples, across different scanners, sequence parameters and scanner software variants will be important to assess generalisability. Future work could also explore whether MRR processing of ULF scans can be optimised by adjusting for dataset-specific properties. In addition, future studies should investigate which anatomical features drive ULF brain-age estimates to assess whether individual tissues, for example, explain more variance in age than others. Finally, while it remains to be determined whether brain-age models trained directly on ULF data may improve performance, this is complicated by the rapid evolution of ULF software. Frequent scanner updates and protocol changes create moving targets that may widen the domain gap, highlighting the need for ongoing benchmarking or the development of approaches that are agnostic to such shifts.

## 5 Conclusion

To our knowledge, this is the first study to investigate brain-age estimation using ULF MRI. By systematically evaluating multiple pipelines, we characterise both the potential and the challenges of extending brain-age modeling to this emerging imaging domain. Our results indicate that ULF can be used to derive accurate and reliable brain-age estimates that approach ground truth and HF benchmarks, supporting the feasibility of brain-age as a biomarker for ULF applications. These findings are consistent with recent studies demonstrating that ULF-derived biomarkers can perform comparably to HF, including work in dementia (Sorby-Adams et al., 2024), multiple sclerosis (Arnold et al., 2022), healthy morphometry (Váša et al., 2025), and normative model centiles (see supplement in Dorfschmidt et al., 2025). We also show that preprocessing choices influence outcomes, emphasising the need for careful benchmarking in future applications. Establishing robustness and clinical utility will require validation in larger and more diverse populations, including clinical cohorts. In this regard, brain-age has the potential to be valuable not only in clinical settings but also as a predictor of health outcomes in healthy cohorts (see also Dorfschmidt et al., 2025). If confirmed more widely, ULF brain-age estimation could provide a practical and scalable tool for clinical decision-making, population research, and long-term patient monitoring, helping to make advanced neuroimaging biomarkers more accessible worldwide.

## Supplementary Material

Supplementary Material

## Data Availability

Data are publicly available https://openneuro.org/datasets/ds006557. Brain-age estimates were obtained using previously published models, with all parameters and analytic steps described in the Methods. For detailed instructions and source code, readers are referred to the original publications and repositories associated with each brain-age model cited in this paper.

## Footnotes

1 https://www.nitrc.org/projects/mricro.

2 https://github.com/james-cole/brainageR as retrieved in September 2023.

3 https://doi.org/10.5281/zenodo.3476365.

4 https://github.com/LemuelPuglisi/SynthBA as retrieved in May 2025.

5 https://github.com/vishnubashyam/DeepBrainNet as retrieved in July 2025.

6 https://github.com/MIDIconsortium/BrainAge as retrieved in July 2025.

7 https://github.com/james-cole/PyBrainAge as retrieved in November 2023.

8 https://surfer.nmr.mgh.harvard.edu/fswiki/FreeSurferWiki.

9 https://surfer.nmr.mgh.harvard.edu/fswiki/recon-all-clinical.

10 https://deep-mi.org/fsqc/dev/.

11 https://surfer.nmr.mgh.harvard.edu/fswiki/SuperSynth.
